# When a Late Metastasis Is Hard to Swallow

**DOI:** 10.7759/cureus.20441

**Published:** 2021-12-15

**Authors:** Catarina Negrão, Rita Sismeiro, Margarida Monteiro, Filipa G Pereira, Marta Jonet

**Affiliations:** 1 Internal Medicine, Hospital Professor Doutor Fernando Fonseca EPE, Amadora, PRT; 2 Anatomic Pathology, Hospital Professor Doutor Fernando Fonseca EPE, Amadora, PRT

**Keywords:** late metastasis, breast cancer, paraneoplastic syndrome, pseudoachalasia, dysphagia

## Abstract

Pseudoachalasia is an uncommon disorder characterised by aperistalsis in the tubular oesophagus and impaired relaxation of the lower oesophageal sphincter (LES). It presents with symptoms and radiologic, endoscopic and manometric findings that mimic idiopathic achalasia. There is a huge spectrum of underlying causes for pseudoachalasia, although malignancy is the most common aetiology.

We report the case of a 70-year-old Portuguese female with a history of breast cancer, submitted to tumourectomy, radiotherapy and hormonotherapy, in complete remission for 16 years, who presented in the emergency department with a two-month history of dysphagia, weight loss, heartburn and nausea. Blood work, body computed tomography (CT) scan, mammography, upper endoscopy, colonoscopy and skeletal scintigraphy did not show any alterations, but barium swallow scan and oesophageal manometry suggested achalasia. She was submitted to oesophageal dilatation with partial symptomatic improvement. Six months later, new onset of dysphonia and worsening of initial symptoms was noticed. A new CT scan revealed unilateral pleural effusion, large mediastinal adenopathy and multiple pulmonary nodules highly suggestive of a metastatic malignancy. Endobronchial ultrasound-guided transbronchial needle aspiration (EBUS-TBNA) from mediastinal adenopathies confirmed the tumoural invasion by a carcinoma, and immunohistochemistry suggested a breast origin. She underwent a nasoendoscopy that revealed bilateral vocal cord paralysis. After chemotherapy was started, symptoms of achalasia completely resolved, and tumour markers, which were increased, have normalised.

The presented case highlights a pseudoachalasia as the first manifestation of a late breast metastasis. It is essential to always have in mind patients’ past history as a key that can help resolve clinical doubts.

## Introduction

Dysphagia is an alarming symptom that requires an efficient evaluation to define the underlying cause and adequate treatment. Achalasia is a rare swallowing disorder that only affects one in every 100,000 people, which results in aperistalsis in the tubular oesophagus and impaired relaxation of the lower oesophageal sphincter (LES) [1]. Achalasia has an insidious onset, and it is characterised predominantly by dysphagia to both solids and liquids, bland regurgitation and chest pain [2-4]. The most common form is idiopathic, and the differential diagnosis includes other oesophageal motility disorders and pseudoachalasia. Pseudoachalasia is characterised by achalasia-like symptoms, and it is usually caused by secondary aetiologies, such as Chagas disease [4], malignancies and infiltrative diseases [5]. Although clinical, radiologic and endoscopic findings mimic those of achalasia, it is essential to define the underlying cause, since the treatment and prognosis depend on it.

## Case presentation

**Figure 1:**
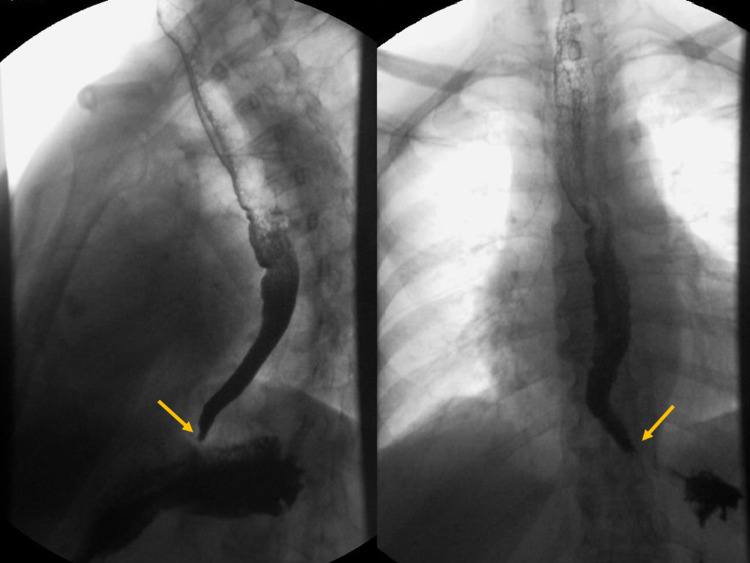
Barium swallow study revealing a bird’s beak sign (arrows).

A 70-year-old Portuguese female presented to the emergency department with a two-month history of dysphagia for solids and liquids, weight loss (about 15% of her total weight), heartburn and nausea. She had a history of left breast cancer treated with tumourectomy, radiotherapy and hormonotherapy (tamoxifen), in complete remission for 16 years. On physical examination, there were no palpable masses, nipple retraction or discharge, changes in the mammary overlying skin and palpable axillary lymph nodes. Macroglossia, microphthalmia, microstomia, skin sclerosis, Raynaud’s phenomenon, sclerodactyly and telangiectasias were not present upon physical examination. She was admitted to an internal medicine ward for investigation. Blood tests, body computed tomography (CT) scan, colonoscopy and skeletal scintigraphy revealed no abnormalities. Mammography and breast echography reported a Breast Imaging Report and Data System (BI-RADS) 2, similar to the previous ones. Upper endoscopy revealed *Helicobacter pylori* gastritis for which she was treated, and there was no evidence of tumour invasion or other infectious aetiology. A barium swallow study showed a bird’s beak sign (Figure 1), and an oesophageal manometry suggested achalasia.

**Figure 2:**
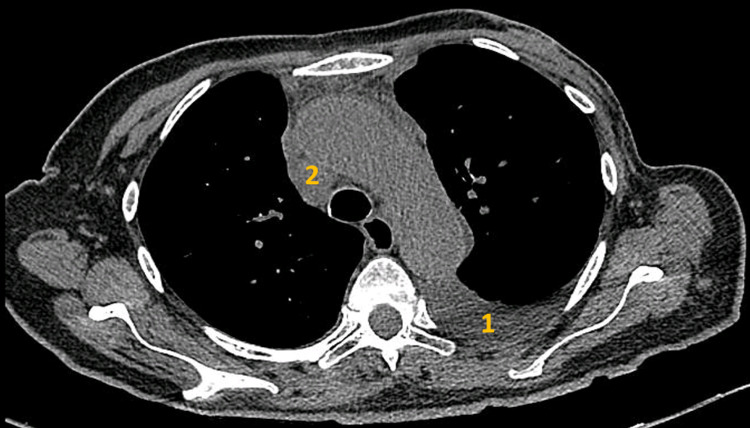
Chest CT showing pleural effusion (1) and mediastinal adenopathy (2).

In order to exclude secondary aetiologies that could suggest pseudoachalasia, further tests were run; autoimmunity panel for scleroderma (antinuclear, anticentromere, antitopoisomerase I and anti-RNA polymerase III antibodies) and nailfold capillaroscopy were negative. Abdominal fat biopsy was negative for amyloidosis. The patient had never been to the American continent, which rule out Chagas disease. The diagnosis of idiopathic achalasia was assumed, and the patient underwent oesophageal dilatation with partial symptomatic improvement. Six months later, the patient noticed worsening initial symptoms associated with dysphonia. A new body CT scan revealed a unilateral pleural effusion, large mediastinal adenopathy and multiple pulmonary nodules, suggesting the possibility of malignancy (Figure 2).

**Figure 3:**
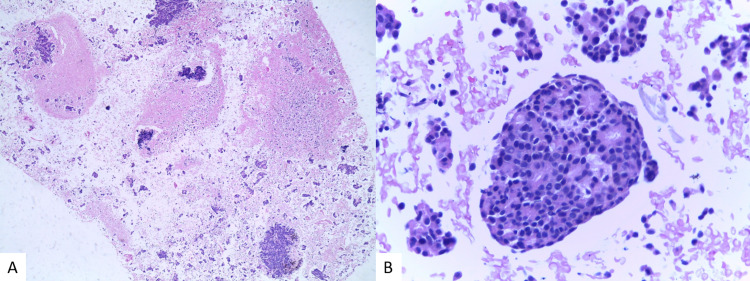
Metastatic carcinoma of the breast (fine needle aspiration) – cell block, haematoxylin and eosin stain (40× (A) and 400× (B)). The cell block section from adenopathy shows moderate cellularity comprising numerous isolated cells and sometimes large neoplastic clusters with cribriform architecture (A). The neoplastic cells have an increased nuclear-to-cytoplasmic ratio, round to oval nuclei, granular textured chromatin, some with prominent nucleoli and finely vacuolated cytoplasm (B).

Endobronchial ultrasound-guided transbronchial needle aspiration (EBUS-TBNA) from mediastinal adenopathies revealed invasion by metastatic carcinoma. The cell block section showed moderate cellularity comprising numerous isolated cells and sometimes large neoplastic clusters with cribriform architecture (Figure 3A). The neoplastic cells had an increased nuclear-to-cytoplasmic ratio, round to oval nuclei, granular textured chromatin, some with prominent nucleoli and finely vacuolated cytoplasm (Figure 3B).

**Figure 4:**
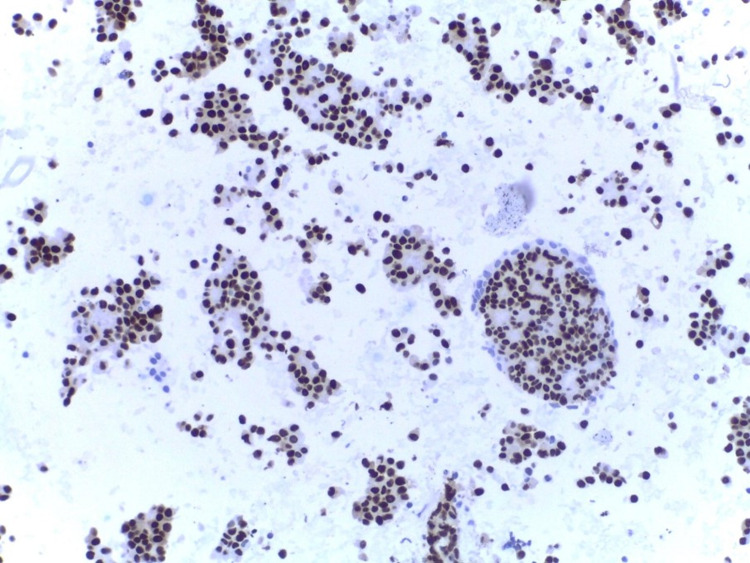
An immunostain for GATA3 shows positive nuclear staining (400×).

The neoplastic cells were strongly positive for cytokeratin 7 and GATA binding protein 3 (GATA3) and negative for TTF1, napsin A, cytokeratin 20, CDX2 and CD117, suggestive of breast origin (Figure 4).

Additional tests revealed a proliferative index (Ki67) of nearly 20%, a hormone receptor-positive tumour (both oestrogen and progesterone receptor were positive) and negative human epidermal growth factor receptor type 2 (HER2). Plasmatic tumour markers were increased (carcinoembryonic antigen (CEA): 47.6 ng/mL; cancer antigen 15.3 (CA 15.3): 40.6 U/mL). As the patient complained of new-onset dysphonia, a nasofibroscopy was performed, which demonstrated bilateral vocal cord paralysis.

Achalasia and dysphonia were admitted as a paraneoplastic syndrome of late metastasis from breast cancer since other aetiologies were ruled out. Unlike paraneoplastic neurologic syndromes, there are no specific antibodies to confirm a paraneoplastic gastrointestinal syndrome. The patient started on hormonotherapy with letrozole, and a clinical improvement was observed with complete resolution of dysphagia and dysphonia, as well as a lowering of the tumour markers after three months of treatment, which supported the paraneoplastic syndrome diagnosis.

## Discussion

Pseudoachalasia is a rare entity. In general, the incidence is 2%-4% amongst patients with suspected achalasia [2]. Up to 70% of cases are secondary to malignancy, with primary malignancy accounting for 54%-70% [5,6] (mostly adenocarcinoma of the oesophagogastric junction or cardia) and secondary malignancy for only 6% [6,7]. Malignancy can cause pseudoachalasia either by direct invasion of the oesophageal neural plexuses (such as in adenocarcinoma of the oesophagogastric junction or cardia) or through the release of unspecified humoral factors that disturb the oesophageal function, especially in patients with small cell lung cancer [8], but this has also been reported in pancreatic cancer [9], pleural mesothelioma [10], multiple myeloma [11], diffuse large B-cell lymphoma [12], metastatic breast cancer [7,13] and cervical carcinomas [14]. Early recognition of malignancy-associated pseudoachalasia is necessary to avoid inappropriate treatment and delay of adequate therapy; however, the differential diagnosis between idiopathic achalasia and pseudoachalasia is very challenging since clinical and diagnostic features, such as radiographic studies, endoscopy and manometry, are often similar in both diseases.

For the diagnosis of achalasia, the assessment of the oesophageal motor function is essential. Endoscopy is the first diagnostic tool to investigate a patient with new-onset dysphagia [2,4]; it detects structural oesophageal and gastric abnormalities, such as tumours. In pseudoachalasia, it is more common to detect a mass, nodularity, irregular mucosa or ulcer [15,16]. Even with normal-appearing mucosa, the difficult passage of the endoscope through the LES suggests pseudoachalasia in contrast to achalasia, in which the endoscope can be passed with delicate pressure through the LES [13,15]. By definition, the manometric finding of aperistalsis and incomplete LES relaxation without evidence of mechanical obstruction solidify the diagnosis of achalasia [2,4].

Facing a normal-appearing oesophagus on endoscopy and new-onset dysphagia, it is important to keep a high index of clinical suspicion to detect the presence of an underlying malignant lesion, especially in advanced age or if there is significant weight loss associated [2,4,7]. Some features can alert for pseudoachalasia, such as an older age of onset and a shorter duration of symptoms with rapidly progressive dysphagia [2,7].

The World Health Organization estimates that, in 2020, breast cancer was not only the most frequently diagnosed cancer (24.5%) but also the most common cause of death in the female population (15.5%) [17]. The factors that predict the risk of recurrence or death from breast cancer include axillary nodal status, tumour size, tumour grade (histologic or nuclear), steroid receptor positivity (oestrogen receptor and progesterone receptor), lymphatic and vascular invasion and metastatic disease [18]. Metastatic breast cancer mainly involves the lungs, bones, brain and liver and rarely the gastrointestinal tract [19,20]. However, it is one of the most frequent origins of oesophageal metastasis [13].

Recognising the range of possible presentations is important for early and accurate diagnosis and treatment. The reported case highlights the importance of excluding secondary aetiologies of achalasia when symptoms persist or relapse, even when the first investigation is innocent, resembling idiopathic achalasia.

In this case, the first careful and detailed investigation did not lead to the diagnosis. Bearing in mind the previous breast cancer diagnosis, maintaining the follow-up and watchful waiting of the patient, we were led to the paraneoplastic syndrome of a late metastasis diagnosis, although more than 10 years have passed since the primary tumour was diagnosed.

In the reported case, a tumour was present, and the other causes for dysphagia and dysphonia, namely, the non-malignant causes of pseudoachalasia, were excluded in two different investigation times. A resolution of gastric symptoms was noticed after tumoural treatment was started. Thus, a presumptive diagnosis of the paraneoplastic syndrome was made; nevertheless, as in the great majority of paraneoplastic syndromes, there are no specific tests to prove it.

## Conclusions

Despite being a rare disorder, it is important to bear in mind that in patients treated for idiopathic achalasia with only partial improvement of symptoms, a persisting wasting syndrome or a history of cancer, pseudoachalasia must always be considered and excluded. A high level of suspicion is needed, since paraneoplastic disorders may precede the primary manifestation of the tumour, relapse or even a late metastasis. With the improvements in cancer treatment, we must be aware of the possibility of late metastasis expressing as paraneoplastic syndromes.

Paraneoplastic syndromes represent a diagnosis challenge since there are no specific antibodies for all the spectrum of these syndromes. Most of the time, the diagnosis of the paraneoplastic syndrome is a presumptive diagnosis, when a tumour is known and other possible causes for the presented symptoms are excluded. A positive clinical response with the treatment targeted to the underlying condition supports the diagnostic hypothesis.
